# Inter-Regional Performance of the Public Health System in a High-Inequality Country

**DOI:** 10.1371/journal.pone.0086687

**Published:** 2014-01-21

**Authors:** Maria Cristina Gramani

**Affiliations:** Insper - Institute of Education and Research, São Paulo, Brazil; Johns Hopkins Bloomberg School of Public Health, United States of America

## Abstract

Previous cross-country studies have revealed a relationship between health and socio-economic factors. However, multinational studies that use aggregate figures could obfuscate the actual situation in each individual region, or even in each individual federal unit, mainly in a developing country that spans a continent and has large socioeconomic inequalities. We conducted a within-country study, in Brazil, of health system performance that examined data in the four perspectives that most strongly affect the performance of public health systems: financial, customer, internal processes and learning&growth. After estimating the interregional health system performance from each perspective, we identified the determinants of inefficiency (i.e., the factors that have the greatest potential for improvement in each region). The results showed that the major determinants of inefficiency in the less efficient regions (N and NE) are concentrated in the perspective of learning&growth (the number of health professionals and the number of graduates with a health-related undergraduate degree) and, in the regions with the best performance (S and SE) the major determinants of inefficiency are concentrated in the financial perspective (spending on health care and the amount paid for hospitalization).

## Introduction

Brazil currently has the seventh-highest gross domestic product (GDP) in the world; however, Brazil is ranked 85^th^ on the health dimension of the Human Development Index (HDI), placing it among developing countries. This discrepancy can be partially explained by Brazil’s vast size and heterogeneous economy among federal units (FUs). For example, “*the southeast region covers only 11% of Braziĺs territory but accounts for 43% of the population and 56% of the gross domestic product*” [1].

Many previous studies have linked socioeconomic inequalities with population health. For example, R Wilkinson reported that “*equal societies are healthier*” [2], and former British Prime Minister Tony Blair stated, “*There is no doubt that the published statistics show a link between income, inequality and poor health… The urgent need is to investigate the role that income plays in promoting health - whether the effects of income come from income itself, or from correlates such as education, wealth, control, or rank*.” [3].

Other studies have analyzed the determinants of health. For example, cross-country comparisons argued that the level of education is a determinant of national health [4]–[5]. One study [4] found differences in morbidity between individuals with high and low educational levels, and the authors suggested that the magnitude of health-related inequality varies according to education among the 11 western European countries considered. Another study [5] used cross-sectional data on 50 developing and transitional countries to show that “*increased public expenditure on education and health care is associated with improvements in both access to and attainment in schools, and reduces mortality rates for infants and children.*” Additionally, a variation across Europe in the magnitude of health-related inequality was associated with socioeconomic status: “*these inequalities might be reduced by improving educational opportunities, income distribution, health-related behavior, or access to health care.*” [6].

Within-country analyses of health disparities have also been performed. In Canada, four of the 12 social health factors most relevant to Canadians were analyzed: early life development, income and income distribution, unemployment and employment security, and housing [7]. More recently, a study conducted an annual examination of a panel in Germany concluding that “*models that take a reductionist perspective and do not allow for the possibility that health inequalities are generated by factors over and above their effect on the variation in health channeled through one of the socioeconomic measures are underspecified and may fail to capture the determinants of health inequalities*.” [8]. Although these studies already reveal the relationships between health and other socioeconomic factors, they only considered developed countries or developing countries as a whole. Studies that use aggregate figures could obfuscate the reality in each individual region, or even each individual federal unit (FU), particularly in a developing country that has continental dimensions and high socioeconomic inequality.

Brazilian health studies have linked socioeconomic factors with the level of health. For example, the acceleration of the decline in undernourished children (in the Northeastern region) between the two studied periods (1986–1996 and 1996–2006) was consistent with accelerated improvement in maternal schooling, water supply and sewage, health care, as well as with the outstanding increase in purchasing power among the poor during the second period [9]. However, despite such progress in Brazil, the social and economic disparities remain unacceptably high, and much effort is still needed to improve the basic living conditions of a substantial portion of the population [10]. For example, only half of Brazilian cities have access to sewage, and only 37.5% of sewage is treated. In the states with the highest rates of hospitalization, access to sanitation services is lower, and vice versa [11]. As a consequence, “*almost half the people in the developing world have one or more of the main diseases or infections associated with inadequate water supply and sanitation.”* [12].

Other studies concerning Brazilian health performance can be found in the literature; some of the studies focus on the efficiency of hospitals or university hospitals [13]–[14], and others focus on the effectiveness of health spending [15]–[18], or, less commonly, on for-profit hospitals [19]. However, these studies mostly focus on the comparisons between hospitals and not on the performance disparity of the public health and hospitals in the different regions of the country.

The Brazilian health system has three subsectors: 1) the public subsector; 2) the private (for-profit and non-profit) subsector, in which services are financed by various public and private funding sources; and 3) the private health insurance subsector. Citizens can use services in all three subsectors [1]. Currently, 60% of the population only has access to the public sector; 61% of these individuals rate the quality of their health as poor or bad, and only 10% assess their health as excellent or good. In regional terms, the southern region offers the best assessment of the country’s public health system: 30% of the residents report that the quality of the system is “excellent” or “good.” The worst ratings come from the northeastern region, where 62% of residents report that the quality of the public health system in their city is “bad” or “terrible” [20].

For the Brazilian public health system, since 1988, each of the 27 FUs is responsible for its own health care through the Unified Health System (*Sistema Único de Saúde;* SUS). According to the Brazilian government (http://portaldasaude.saude.gov.br), the SUS lists the same responsibilities for all FUs; the SUS “*covers procedures from simple health procedures to outpatient organ transplants, ensuring fullaccess and universal and free coverage for the entire population*”. However, the decentralized management of the health system may lead to performance variations between FUs.

The main aim of this paper was to investigate from various perspectives the performance of the public health system among the federal units, disaggregating the factors that have a significant influence on the health sector and identifying potential areas of improvement for each FU in the country (which are likely to be different for each FU).

## Methods

### Definitions of Health Perspectives and their Respective Variables

Studies that evaluate the performance of health systems typically use variables directly related to health care, such as the number of health professionals, costs, number of inpatients, patient life expectancy at birth, and others [21]–[24]. In general, these studies do not include environmental factors such as education, income, employment, and sewage treatment that also have an influence on health performance.

To categorize all of the relevant variables (directly related to health and non-directly related to health), we use the classical balanced scorecard method (BSC), originally developed by Kaplan and Norton [25], which has been adopted by a wide range of health care organizations. According to the Balanced Scorecard Institute, “*The Balanced Scorecard is a strategic planning and management system used to align business activities to the vision and strategy of the organization, improve internal and external communications, and monitor organizational performance against strategic goals.*”

This method allows managers to look at the health systems from four important perspectives: financial, customer, internal processes and learning & growth. The meaning and integration of these four perspectives defines the BSC system, which must be connected to the strategic goals of the organization. Thus, the BSC model differs from other measurement systems in that it requires relations between the perspectives [26].

A comprehensive review shows that BSC has been introduced across all areas related to healthcare, such as hospitals, health care systems, university medical/health departments, long-term care facilities, mental health centers, pharmaceutical care and health insurance companies [27]. The authors concluded, among other things, that “*perspectives that are commonly added to balanced scorecards for health care organizations include quality of care, outcomes and access*”. Other studies have also used BSC perspectives in health systems [28]–[30], although the relationship between health and other environmental factors were not pondered, or just one district was considered (i.e., the focus was not on the comparison between the regions or federal units within a country). More recently, a concept derived from the BSC was developed as a national scorecard to assess the New Zealand health system, but again, it was not the purpose of the paper to include indicators related to high socioeconomic inequalities [26]. In this way, Kaplan and Nortońs four perspectives (financial, customer, internal process and learning) appear to be the template for implementations in healthcare, no matter how they were modified in practice [31]. Therefore, “*despite the increased adoption of the BSC methodology by numerous business organizations during the last decade, limited case studies concern non-profit organizations (e.g., public sector, educational institutions, healthcare organizations, etc.)*” [32]. Thus, BSCs are still in an evolutionary stage in health care settings, and strategy mapping is not yet common [31].

One advantage of using the BSC methodology to categorize the aforementioned health perspectives is that it allows for the consideration other factors that significantly impact health systems, such as education, income, employment and sewage treatment. These four non-health factors are very significant when considering the welfare of citizens in Brazil and are critical to improving national care. For example, an analysis of the causes and consequences of improvements in life expectancy across Brazilian municipalities shows that improvements in education, access to water, and sanitation seem to be important determinants of the dimension of changes in life expectancy not correlated with income [16].

Thus, the significant variables used to analyze the public health system in Brazil are divided into four perspectives. Table 1 illustrates the general vision of the public health system in Brazil (SUS) and the strategic objectives for each BSC perspective, as derived from some literature references [31]–[36].

**Table 1:** Vision, BSC-perspectives and strategic goals for SUS.

Vision: *covers procedures from simple health procedures to outpatient organ transplants, ensuring fullaccess and universal and free coverage for the entire population * [*33*] *.*	
Perspectives	Strategic goals
**Financial**	To spend in an efficient manner in order to assist the population [34]
**Customer**	To ensure the quality of care [32]
**Internal process**	To ensure the primary resources are used in order to take into account particular environmental aspects of the region. [35]
**Learning& growth**	To ensure the capacity of the organization for the long-term run (human capital and/or information capital and/or organization capital) [31]

Following the strategic goals presented in Table 1, we propose a group of indicators as detailed below (a code for each variable is designated in parenthesis).

#### 1. Financial perspective

This perspective indicates whether the public health spending is being done in an efficient manner in order to assist the population. For this, we use the two variables as the financial resources (inputs): *spending on health care* (I1), or spending on initiatives and public health services, and the *average amount paid for hospitalization* (I2). Because we investigated public health systems (i.e., not-for-profit organizations) instead of considering financial variables as outputs (such as revenue, profit, etc.), we use the *number of available beds* (O1) as a proxy for the availability of health service delivery, since “*a greater number of hospital beds suggest greater availability of inpatient health services.*” [12]. Thus, this perspective will indicate which regions perform better in terms of the availability of inpatient health services and using fewer financial resources.

#### 2. Customer perspective

This perspective indicates whether the capacity to serve patients meets the needs of the citizens with high quality patient care. In this category, the *number of beds available* (I3), used as a proxy for the capacity to serve the patients, is the resource (input) which impacts the *number of inpatients* (O2) and *life expectancy at birth* (O3). The link between the *number of beds available* and the *number of inpatients* is based on “measuring the capacity for serving patients”. The link between the *number of beds available* and *life expectancy at birth* is based on the “quality of patient care”. The *life expectancy at birth* is also used as a measure of health attainment in the literature [37]–[39]. For Health Systems 20/20 (2012, p.101), this is a common indicator of the quality of the health system, as countries with low life expectancy generally are perceived as having weaker health systems than those with longer life expectancies [12]. Accordingly, this perspective will show which areas perform better in terms of the *number of inpatients* served with high quality health care (*life expectancy at birth)* using fewer resources.

#### 3. Internal processes perspective

Health in Brazil is a multifactorial issue that covers society, economics and public policy [36]. This perspective tries to capture particular environmental conditions of the federal units in order to explain the health performance in the context of a system. We use as inputs *health coverage plans* - I4 (a proxy for the population served by care), *customer care performance* - I5 (a proxy for the quality of care) and *sewage treatment* - I6 (a proxy for the environmental condition), which affect *education* (O4), *average income* and *unemployment rates* (O5). Thus, this perspective attempts to demonstrate that higher levels of education, income, and employment are related to better environmental conditions and health care.”

#### 4. Learning & growth perspective

This perspective aims to ensure the capacity of the human capital of the health system long-term. This perspective intends to link *level of education* (I7), *unemployment rate* and *average income* (I8) with the *number of health professionals* (O6) and the *number of graduates with a health-related undergraduate degree* (O7). That is, this perspective captures the fact that greater numbers of health professionals and graduate students are attracted to regions with higher levels of education, income and employment.

As can be noted, some variables can be used in more than one perspective because the BSC perspectives are interlinked; for example, the *number of beds available* can be viewed as a product in the financial perspective or as a resource in the customer perspective. Table 2 presents the conceptualization of all the variables used in the four perspectives.

**Table 2:** Conceptualization of all variables used in BSC-perspectives.

Variable	Conceptualization
**Spending on health** **care per capita**	Public health expenditure per capita, according to the sphere of government, in a particular geographical area for the year in question.
**Average amount paid for** **hospitalization in the public health** **care system**	Average value of inpatient care in the public health system (SUS), by specialty, in a certain geographical area in the current year.
**Number of beds available per** **1,000 inhabitants**	Number of public and private hospital beds, linked or not to the public health system (SUS), per thousand inhabitants in a given geographical area in the current year.
	
**Number of inpatients per** **100 inhabitants**	Average number of hospitalizations paid by the public health system (SUS), per 100 inhabitants, the population living in a given geographic area in the current year.
**Life expectancy at birth**	Average number of years of life expected for a newborn, the pattern of mortality within a population residing in a given geographic area in the current year.
**Health coverage plan**	Percentage of the population covered by insurance plans and supplementary health care in a given geographical area in the current year.
**Population served by sewage** **treatment**	Percentage of the population of residents who has sewer waste by connecting the home to the collecting system or septic tank, in a certain geographical area in the current year.
**Total population**	Total number of residents and their relative structure in a given geographical area in the current year.
**Population with more than** **8 years of education**	Percentage distribution of the resident population between 18–24 years old with 8 to 10 years of study in a given geographical area in the current year.
**Average income per capita**	Ratio income of the top fifth of the income distribution (richest 20%) to the income of the bottom quintile (poorest 20%) in the population residing in a given geographic area in the current year.
**Unemployment rate**	Percentage of economically active resident population that is without work during the reference week in a certain geographical area in the current year.
**Number of health professionals** **per 1,000 inhabitants**	Number of health professionals per thousand inhabitants according to categories in a given geographical area in the current year.
**Number of graduates with a** **health-related undergraduate** **degree**	Number of graduates of undergraduate health by higher education institutions in a specific geographic area depending on the year considered.

**Source**: Basic Indicators for Health in Brazil 2008**–2^nd^ Edition** (http://tabnet.datasus.gov.br/tabdata/livroidb/2ed/matriz.pdf).

Differently from studies in developed countries with socioeconomic equity (and, in general, universal and free access to hospital care), where the improvements are more related to performance and health, such as obesity, alcohol consumption, screening, preventive care, etc. [26], [40], the proposed group of indicators presented above tends to include financial access to care barriers, unmet health care needs, proximity of population to health care, socioeconomic aspects, etc.

### Source of Data

The cross-sectional data used in this study are publicly available and were collected from Datasus, an SUS database (http://tabnet.datasus.gov.br). Datasus is a government information center that measures the health of the population. This database collects various indicators, including demographic and socio-economic information, financial resources, access to health, and sanitary services. Among these indicators are many variables that are directly and indirectly related to health.

The data for all of the variables mentioned in the previous section were collected from all 27 Brazilian FUs in 2008, 2009, or 2010 (the most current data available), and the descriptive statistics are shown in Table 3.

**Table 3:** Descriptive statistics for all variables.

Variables - Year	Mean	Standard Deviation	Median	Min	Max
Spending on health care per capita(in Real) - 2010	632.7	124.1	639.5	397.6	877.9
Average amount paid for hospitalizationin the public health care system(in Real) - 2010	839.2	194.4	826.5	516.5	1,172.3
GDP per capita (in Real) - 2009	14,600.2	8,980.5	13,269.4	6,051.5	50,438.4
Number of beds available per 1,000inhabitants - 2009	2.1	0.3	2.2	1.6	2.9
Number of inpatients per 100inhabitants - 2010	6.1	0.9	6.1	4.2	7.5
Life expectancy at birth - 2010	72.7	2.3	72.4	68.0	76.0
Health plan coverage (percentageof the population) - 2008	19.4	9.1	15.1	6.0	40.1
Population served by sewagetreatment - 2010	4,536,540.4	7,573,338.6	1,798,600.0	148,464.0	37,209,765.0
Total population - 2010	7,065,029.6	8,410,048.7	3,514,952.0	450,479.0	41,262,199.0
Population aged between 18–24 with8 to 10 years of education - 2009	218,302.3	230,303.3	120,457.0	13,276.0	1,055,958.0
Average income per capita(in real/month) - 2010	675.2	281.2	575.4	348.7	1,665.4
Unemployment rate - 2010	250,928.9	319,917.2	131,689.0	14,134.0	1,549,972.0
Number of health professionals (doctors)per 1,000 inhabitants - 2010	1.5	0.8	1.2	0.5	3.6
Number of graduates with a health-relatedundergraduate degree - 2010	3,593.8	5,334.8	1,863.0	65.0	24,705.0

### Quantitative Analysis: Data Envelopment Analysis

“*… while BSC provides a vast array of individual quantitative indicators, it does not provide for consolidated performance values, either for the individual perspectives or for their consolidation.*” [34]. More recently, “*When many perspectives are considered in the BSC framework with several measures in each perspective, the ability of managers to comprehend the huge volume of information becomes limited*.” [30].

According to the World Health Organization (WHO), “*One health system differs from the others in structure, quantity and kinds of resources utilized and the outcomes attained. Nevertheless, health authorities yearn for the same objectives, which are based on values like: good health for the entire population, responsiveness and fairness in financing*.” [41]. In this manner, some authors seek quantitative methodologies to consolidate the performance values of each perspective in the BSC approach. Data envelopment analysis (DEA) is one method that can be used for consolidation purposes.

**Figure 1:**
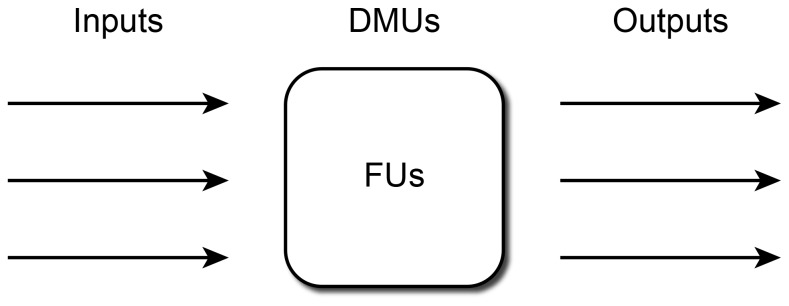
Illustration of DEA.

DEA is a non-parametric linear programming technique that can be used for consolidation purposes by comparing Decision-Making Units (DMU) that use the same inputs to generate the same outputs but differ in quantity (Figure 1). The DMU with the highest ratio of outputs to inputs is deemed the top performer [42].

The decision-making units considered in this study are the 27 FUs, and DEA provides a quantitative method for assessing their relative performance from each BSC health perspective. Although this method does not identify the best possible DMU performance, it does identify which DMU is performing the best among the DMUs considered. Moreover, this methodology also reveals how inefficient DMUs may reach maximum efficiency.

**Figure 2:**
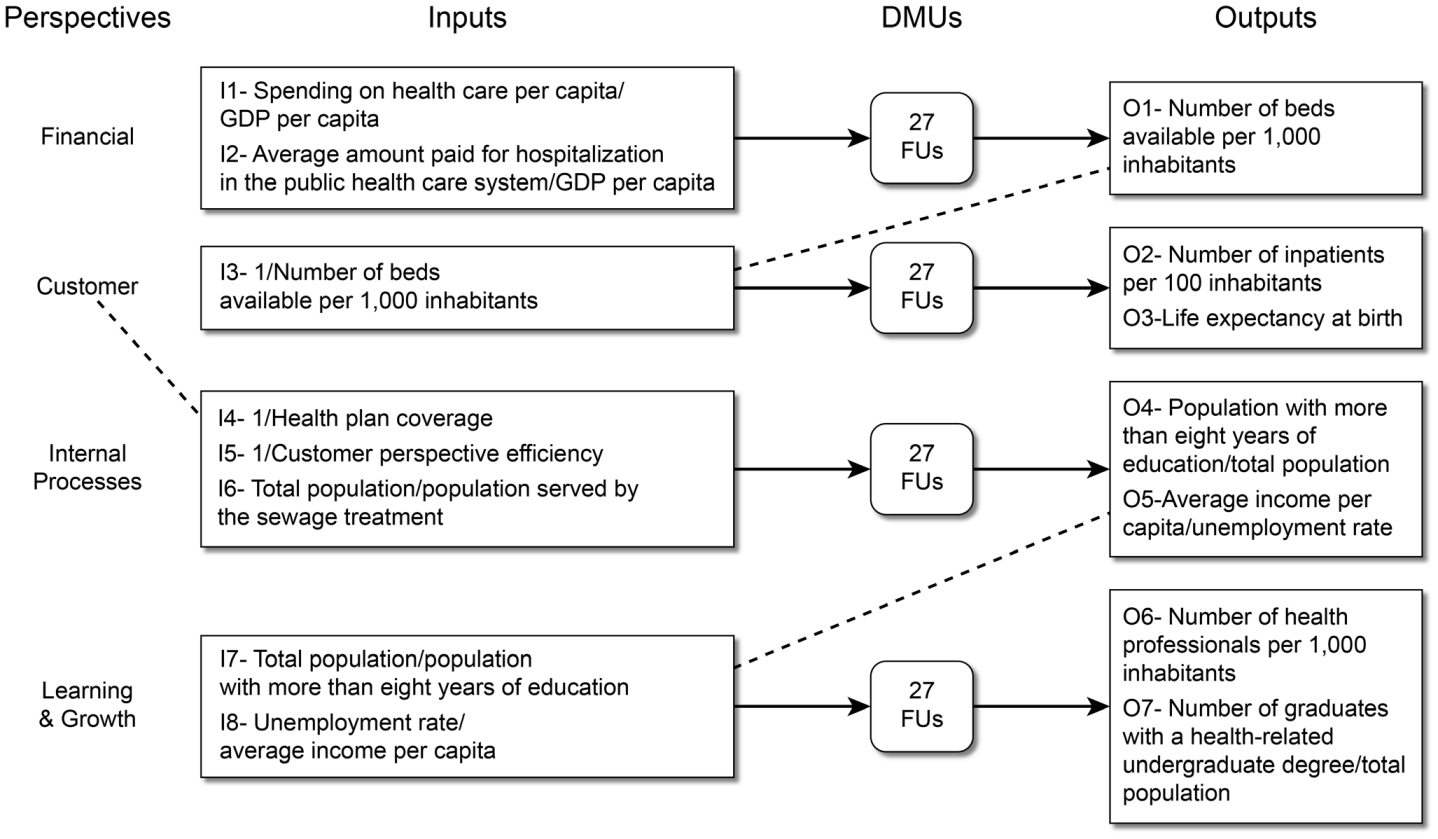
Integrated BSC-DEA model for the SUS.

We propose an integrated BSC-DEA model that evaluates the performance of the FUs from each of the four BSC perspectives, as shown in Figure 2. We believe that the BSC approach can offer a useful framework to structure several interconnected DEA models [43].

The dotted lines in Figure 2 show the following dependencies among the perspectives: the outputs of the *financial perspective* are the inputs for the *customer perspective;* the efficiency score from the *customer perspective* is an input for the *internal process perspective*; and finally, the outputs of the *internal process perspective* are the inputs for the *learning & growth perspective*. This integration follows the DEA network approach where the outputs of one BSC perspective are considered as inputs for the following perspective [44] and reflects that the objectives in one perspective emphasize the objectives of other perspective. The integration of BSC and DEA was also proposed for assessing the performance of primary care trusts in the UK. The authors used six perspectives for the BSC considering different sets of inputs and outputs for the DEA model in each perspective and, using variables directly related to health in all perspectives, i.e., no socioeconomic variable was considered [30].

It is important to mention that many variables (inputs and outputs) may reflect several efficient DMUs, thus some components found in other health BSCs (as variables directly related to diseases) were not considered in the proposed model. To achieve a reasonable level of discrimination, the number of units (DMUs) should be at least 2×*S*, where *S* is the product of the number of inputs and number of outputs [45]. Also, we used the variable returns to scale (VRS) for the DEA model for all of the perspectives because we determined that the increases in the inputs and outputs are not proportional. Furthermore, only the financial perspective considers the input-oriented model; the other three perspectives consider the output-oriented model.

## Results

### Health Performance Comparison

**Figure 3:**
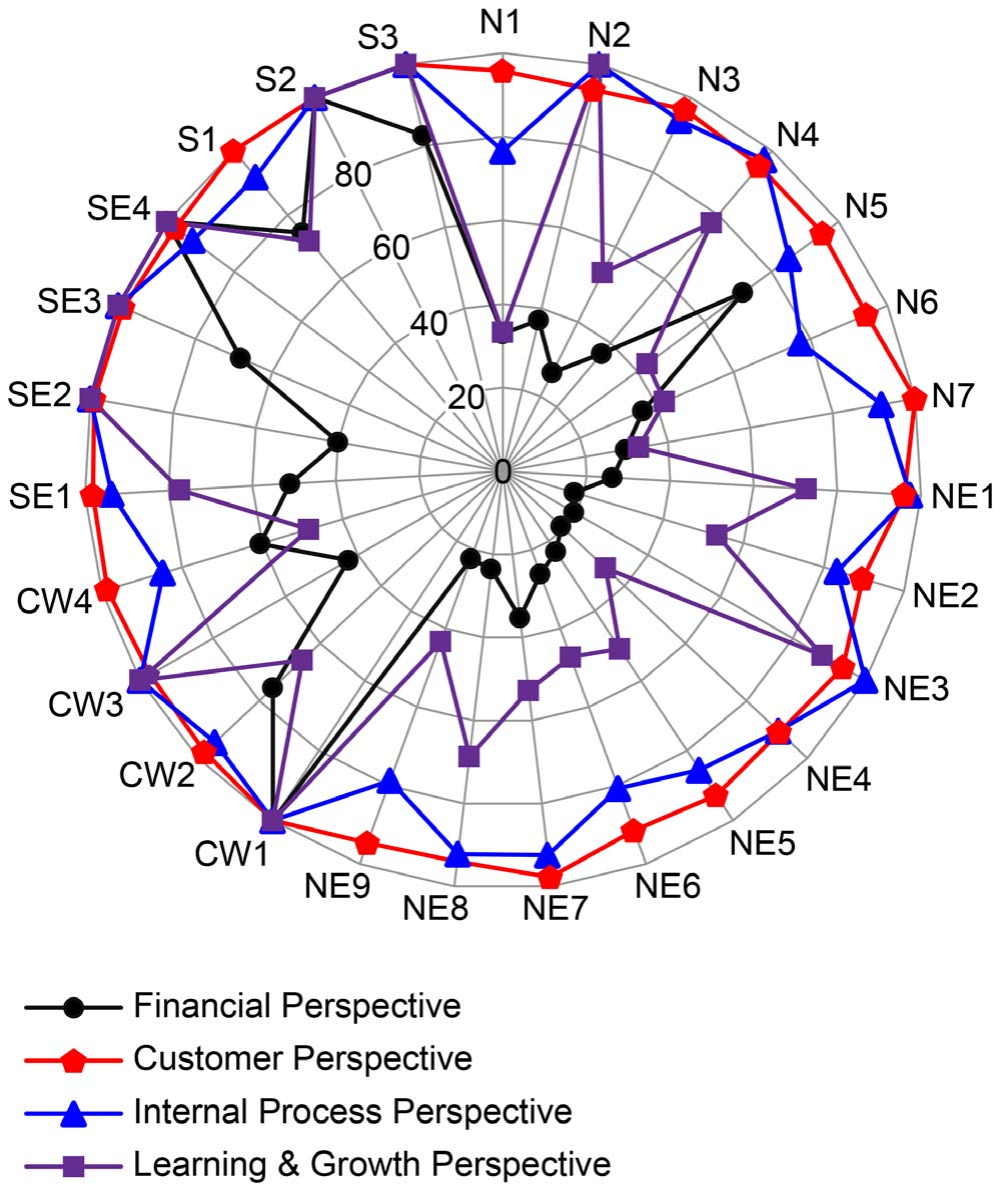
Public health system performance.

Figure 3 presents the results of the four DEA models generating the relative public health performance from each BSC perspective. The FUs N1 to N7 are located in the northern region of the country; NE1 to NE9 are located in the northeastern region; CW1 to CW4 are located in the central-west region; SE1 to SE4 are located in the southeastern region; and S1 to S3 are located in the southern region.

Figure 3 shows three main results: first, there are differences in performance associated with different perspectives: the FUs perform best from the *customer perspective* and perform worst from the *learning & growth* and *financial perspectives*. Second, there are differences in performance between the FUs: the FUs from the S and SE regions are clearly superior to the FUs from the N and NE regions. And finally, all of the FUs from the S region are 100% efficient from the *customer perspective* (i.e., this region could serve as the benchmark for this perspective).

### Determinants of Inefficiency and Potential Improvements

This section describes the causes of inefficiency in each region and discusses potential areas for improvement.

**Figure 4:**
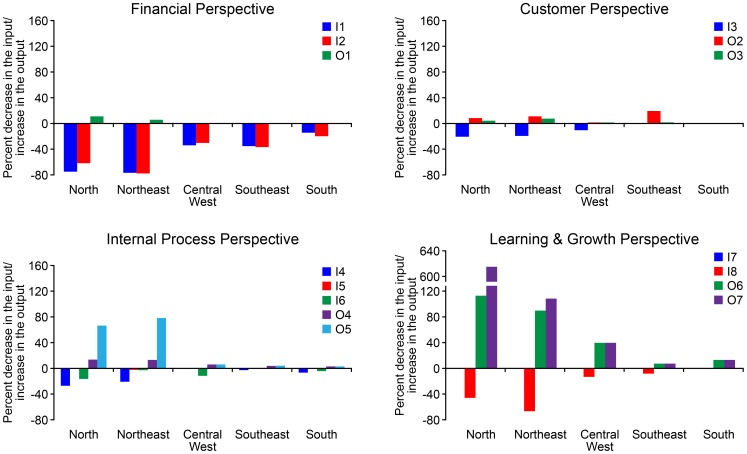
Potential improvements.

Figure 4 indicates the extent to which each variable in each region must improve to reach maximum efficiency. The vertical axes represent the percentage decrease in the input or the percentage increase in the output that must be achieved to reach maximum efficiency (note that DEA models attempt to minimize the inputs and maximize the outputs [45]). For example, from the *financial perspective*, the N region must decrease input I1 (*spending on health care per capita/GDP per capita*) by 74%, decrease input I2 (*average amount paid for hospitalization/GDP per capita*) by 60%, and increase output O1 (*number of beds available per 1,000 inhabitants*) by 11% to make this region 100% efficient.

In addition, regions with better performance have relatively less to improve. For example, the S region has nothing to improve in customer perspective because this region is much better than the others in this perspective.

It is noteworthy that the N and NE regions have the greatest potential improvement figures compared with the other three regions; this result is consistent with the results that show inferior efficiency in the N and NE regions (Figure 3).

It is also important to note that the “strongest” determinants of inefficiency from each perspective can differ between regions. The two strongest determinants of inefficiency in each region can also be observed in Figure 4. This figure shows the differences in needs, particularly between two groups of regions: N/NE and SE/S. For the N/NE regions, the major potential improvements are concentrated in the *number of health professionals-O6* and the *number of graduates with a health-related undergraduate degree-O7* (the outputs from the *learning & growth perspective*), whereas the majority of the potential improvements for the SE/S regions are related to *spending on health care per capita-I1* and the *average amount paid for hospitalization in the public health care system-I2* (the inputs from the *financial perspective*). The majority of the potential improvements for the CW region are almost equally divided between two perspectives, *financial* and *learning & growth.*

## Discussion

We found evidence that indicates the importance of separating performance evaluations of the public health system into four perspectives. The results clearly show that if we consider only one general perspective, we will merely obtain an overall health classification, and a number of key indicators will likely be overlooked. Furthermore, the information obtained can help government decision-makers determine which resources will truly affect the performance of the health system in each region of the country.

We found that only one FU excels in all four perspectives (CW1, that is the smallest federal unit of Brazil - the District Federal - and contains 31 administrative regions, including the Brazilian capital city), but unlike the results obtained by other researchers [30], we found that regions that perform well from one perspective also perform well from the other perspectives. The authors [30] did not find a single HA that performed consistently well in terms of all the six perspectives; moreover, they concluded that an HA that performs well from one perspective does not necessarily perform well from the other perspectives. The reason for this difference could be the high socioeconomic inequality in Brazil, where richer regions perform better.

In addition, our results indicated that the difference between the performance of the N/NE regions and the S/SE regions is remarkable, and it is clear that the N/NE regions must improve more than the other regions to achieve efficiency.

As reported for Europe [6], the inequalities in Brazilian health care are also highly associated with socioeconomic status (Table 4). The regions that perform better in terms of education (evaluated using data from the latest version of the Program for International Student Assessment – PISA) and economic production (evaluated using GDP data) are also superior in terms of health care.

**Table 4:** Correlations between the efficiency indexes (for each region) and PISA/GDP.

	Financialperspective	Customerperspective	Internal processesperspective	Learning & growthperspective
PISA - 2009	0.98	0.96	0.99	0.96
GDP - 2009	0.88	0.89	0.88	0.84

Nevertheless, the major determinants of inefficiency (i.e., the factors that most need improvement in each region) differ by region. The results show that the *learning & growth perspective* deserves more attention in the N/NE regions because of the lack of health care professionals (Table 5). The results suggest that the N/NE regions need more incentives to attract and retain health professionals in their FUs. These results can be partially explained because of the concentration of residency programs in the S/SE region. These residencies could be one of the factors that promote the unequal geographic distribution of physicians, while the N/NE regions stand out as attractive to migrant doctors [46].

**Table 5:** Number of health professionals per 1,000 inhabitants, demographic density and the number of inhabitants older than 60 years old in Brazil.

	Number of health professionalsper 1,000 inhabitants	Demographic density	Elderly (60 or older)
North	0.98	4.12	7%
Northeast	1.19	34.15	10%
-Central-West	1.99	8.75	9%
Southeast	2.61	86.92	12%
South	2.03	48.58	12%

Source: IBGE/BRASIL, 2010.

Other factors have the greatest impact on efficiency in the S/SE regions, namely, *health care spending* and the *amount paid for hospitalization*, which are both related to the financial perspective. The first factor could be related to the greater demographic density of these regions and the high in-migration from other regions; the latter, which is related to the high spending in these regions, could be in part explained by the large elderly population in the S/SE regions, as shown in Table 5. A comparison of the inpatient care of the elderly between Brazil and India shows that there was no evidence of inequality in Brazil in either the receipt or length of stay by income per capita, but the higher educated individuals had longer stays in hospital in Brazil [47]. As the S/SE regions have the highest level of education in the country, this could also explain the high spending on hospitalizations by the elderly population. Thus, in these regions (S/SE), the determinants of inefficiency are mostly related to the financial perspective.

Finally, in both the N/NE and S/SE regions, the population experiences problems with access and substantial delays in receiving care. In the N/NE regions, this problem can be attributed to the lack of health care professionals, and in the S/SE regions, it is caused by the lack of funding for increasing and/or improving the infrastructure. It is clear that socioeconomic inequalities affect health care performance, and most policies are general in scope aim to target the entire country. This paper aims to help policy makers when choosing programs and health policies for the different regions of the country.
